# Hepatitis B vaccination at birth: science, myths, and the safety net imperative

**DOI:** 10.3389/fimmu.2026.1770223

**Published:** 2026-03-17

**Authors:** Rajesh K. Gupta

**Affiliations:** Biologics Quality & Regulatory Consultants, LLC, North Potomac, MD, United States

**Keywords:** hepatitis B vaccine birth dose, vertical and horizontal tansmission, myths and facts, risk in infants, chronic hepatitis B, publich health policy

## Introduction

Hepatitis B virus remains one of the world’s most consequential infectious pathogens, responsible for an estimated 254 million chronic infections with 1.1 million deaths in 2022 (1), primarily from cirrhosis and hepatocellular carcinoma. Currently, there is no complete cure for hepatitis B, and there is a stigma associated with chronic hepatitis B. The recommendation for universal Hepatitis B vaccination at birth is among the most successful public health achievements in modern history. Its implementation, pioneered in the United States (2–4) and adopted globally, along with catch-up vaccination for unvaccinated children (5), has led to a near-elimination of pediatric hepatitis B infection in the US and many other countries. Yet, decades later, this cornerstone of preventive medicine continues to be challenged by persistent myths, controversies, and debates that often overlook the core scientific evidence. Frequently, debates in health policy, parental communities, and even clinical settings continue to be shaped by misinformation, particularly in the age of social media. This commentary aims to re-anchor the conversation in science, address persistent myths, and contextualize the public health imperative for timely vaccination. This perspective examines the scientific rationale behind the birth dose, disentangles persistent myths, and underscores why timely vaccination remains essential to public health.

## The science: zero tolerance for chronic infection

The rationale for the Hepatitis B vaccine birth dose stems from the unique pathology of the Hepatitis B virus in infancy and the marked differences in its transmission dynamics between adults and infants. When an adult is infected with the Hepatitis B virus, they have a 90% chance of clearing the virus, and only 5-10% of adults will develop chronic hepatitis B. However, hepatitis B virus persists as stable, covalently closed circular DNA (cccDNA) in the hepatocyte nuclei of anyone ever infected, acting as a “mini chromosome” reservoir for life (6, 7). This latent cccDNA can cause severe viral reactivation if the host’s immune system is suppressed by chemotherapy, transplantation, or other immunomodulatory therapies (6, 7). Conversely, 90% of infants infected at or around birth will develop lifelong chronic infection, often silently, with lifelong consequences. It is this devastating difference in the virus’s pathogenicity among adults and infants that makes early infection so catastrophic.

Chronic Hepatitis B infection is the leading cause of liver cirrhosis and hepatocellular carcinoma worldwide. A baby infected at birth has approximately 25% chance of dying prematurely from these complications (8). Hepatitis B vaccine has therefore been rightly considered the first anticancer vaccine, and is, in effect, a “prenatal cancer prevention” measure, protecting infants years before they are at risk of sexual or injection-related exposure.

The biological vulnerability in early life, coupled with high maternal viral loads, creates a narrow window of prevention. For infants born to HBsAg-positive mothers, the combination of the hepatitis B vaccine and Hepatitis B Immune Globulin (HBIG) administered within 12 hours of birth constitutes post-exposure prophylaxis (8). This dual action is 85–95% effective at preventing transmission, a life-saving intervention that cannot be delayed. Pregnant women with high HBV DNA levels are recommended to receive antiviral therapy during pregnancy to further reduce/eliminate the risk of perinatal transmission (9).

To effectively control and eliminate the Hepatitis B virus, the focus must remain on early detection and prevention through maternal screening and universal infant vaccination, recognizing the disease’s root cause in infancy rather than its later manifestations in adulthood.

## Addressing persistent myths

### Myth 1: The vaccine is unsafe for newborns

• Fact: Excellent Safety Track record for the vaccine

The Hepatitis B vaccine is among the most studied vaccines in history, with over 1 billion doses administered globally. Safety reviews by the World Health Organization (WHO) and major national bodies have repeatedly confirmed its excellent safety record. Adverse events are overwhelmingly minor (soreness, low-grade fever), and large-scale epidemiological studies have consistently refuted claims linking the vaccine to severe long-term outcomes.

• Fact: The vaccine is Safe at birth or 2 months

A recent comprehensive review of published studies found no evidence that delaying the birth dose improves safety or effectiveness in the U.S. population (10).

### Myth 2: Hepatitis B is a disease of adolescents and adults, transmitted through sex, sharing needles by drug users, and blood transfusion

• Fact: Transmission Occurs Beyond Sex and Transfusions

While sexual contact and contaminated injections (including transfusions) have been major routes of spread among unvaccinated adults, they are not the leading cause of the global burden of chronic disease. Every unvaccinated infant, child, and adult must take the hepatitis B vaccine to prevent further infections. In countries like the U.S., blood transfusions are no longer a significant route of transmission due to rigorous, mandatory testing of the blood supply.

• Fact: Hepatitis B is Primarily a Perinatal Infection

The most significant public health concern is not adult transmission, but mother-to-child (perinatal or vertical) transmission, which occurs during or shortly after birth. Most people living with chronic Hepatitis B infection today acquired it as an infant. The high rate of chronicity means that the disease burden (cirrhosis and liver cancer) experienced by adolescents and adults is primarily the result of an infection that occurred 20 to 40 years earlier, in infancy.

• Fact: Horizontal transmission in early childhood – a significant route in Endemic settings

In early childhood, the virus can spread through non-sexual, non-blood-transfusion routes via close household contact. This occurs when an uninfected child comes into contact with the blood or body fluids of a chronically infected household member (e.g., sharing personal items contaminated with microscopic blood, such as toothbrushes or razors). Before universal vaccination, this accounted for a large portion of childhood cases.

### Myth 3: Perinatal transmission can be fully controlled by maternal screening alone

• Fact: In practice, incomplete or late antenatal testing, missed diagnoses, unrecognized maternal infection, and the narrow window of labor and delivery render selective strategies insufficient. Based on US claims data, approximately 12–18% of pregnant women did not receive recommended antenatal hepatitis B (HBsAg) screening. Data from 2014 showed that 12.3% of commercially insured and 16.4% of Medicaid-enrolled women did not receive testing, and later studies (2015–2020) confirmed that roughly 14.6% of pregnant individuals missed screening, risking vertical transmission (11). A universal hepatitis B vaccine within 24 hours of birth is therefore indispensable, because the most consequential infections occur before a child’s first birthday.

• Fact: The vaccine ensures the protection of babies due to gaps in health care during pregnancy

This view emphasizes the crucial role of the Hepatitis B vaccine given early in life (before 2 months of age) as a critical public health safety net. Despite mandatory prenatal screening, maternal status is often unknown at the time of birth in a significant percentage of cases due to lack of prenatal care, late presentation, or logistical documentation errors. In regions with intermediate-to-high prevalence, up to a third of mothers with chronic Hepatitis B infection are unaware of their status.

### Myth 4: The vaccine is unnecessary if the mother is Hepatitis B negative

• Fact: The vaccine protects against Horizontal transmission.

The Hepatitis B vaccine given early in life also protects against horizontal transmission – the acquisition of the virus after birth from an infected, often asymptomatic, household or community member. Before universal vaccination, community exposure accounted for a substantial portion of pediatric infections. The Hepatitis B virus is highly resilient and can remain infectious on surfaces for over seven days. Delaying the vaccine leaves the baby unprotected during the most vulnerable early months of life against a silent public health threat.

Selective vaccination based on maternal testing alone risks preventable infections.

## Controversies surrounding the timing of the first dose

One recurring controversy concerns whether vaccination can be deferred until two months of age in infants born to hepatitis B–negative mothers.

• Maternal antibodies don’t interfere with vaccine immunogenicity

Concerns have been raised that transplacentally acquired maternal antibodies may interfere with the immunogenicity of certain vaccines administered early in life by masking epitopes or accelerating antigen clearance. This has been demonstrated with live attenuated viral vaccines, such as measles, in which maternal antibodies reduced vaccine effectiveness, necessitating delayed administration (12). However, this does not hold for the hepatitis B vaccine, for which it has been shown that neonates develop protective antibody responses and durable immune memory even in the presence of high maternal antibodies (13) and HBIG, given to babies born to hepatitis B-positive mothers.

• Scientifically, a Vaccine at birth or at 2 months does not affect the immunogenicity

There are approximately 3.6 million births in the US (2023 data), with almost 95% occurring in women under 40 years old (14). Most of these women should have been vaccinated against hepatitis B under the policies – universal vaccination of newborns with the hepatitis B vaccine since 1991 (3), catch-up vaccinations for adolescents around 11–12 years in 1995 (4), and for all children in 1999 (5). However, CDC reported that self-reported vaccination coverage (≥3 doses) was only 40.3% among adults aged 19–49 years in 2018 (15). With maternal antibodies from immunized mothers, there should be almost no risk of hepatitis B to babies under 2 months, justifying delaying the hepatitis B vaccines to babies born to hepatitis B-negative mothers.

• Simplicity and ease of vaccination at birth

It is much easier and more effective, from a public health standpoint, to have a single vaccination procedure for hepatitis B – vaccinate every baby at birth - than to rely on a complex, risk-based system that requires perfect screening and coordination for every mother.

Based on these facts, it does not seem to matter much if the babies born to hepatitis B-negative mothers are vaccinated at birth or at 2 months. The final protective antibody level (immunogenicity) may be similar whether the first dose is given at birth or later. The unnecessary debate and this controversy are creating confusion among parents and may generate vaccine hesitancy, which is already a problem.

## Conclusions

The global experience with Hepatitis B vaccination is one of the most compelling success stories in preventive medicine. However, its full potential can only be realized when the birth dose is administered universally and on time. In an era where misinformation often outpaces public health messaging, reaffirming the evidence behind this simple, life-saving intervention is both urgent and essential.

The science is clear; the myths are unfounded; controversies are unnecessary; the facts point to one conclusion: Hepatitis B vaccination at birth to babies saves lives. Importantly, timely vaccination protects infants not only from vertical transmission but also from horizontal transmission in early childhood – an underappreciated but significant route in many endemic settings.

The universal hepatitis B vaccination for babies at birth is a proven public health policy forged by the failure of risk-based strategies. It is a simple, high-efficacy intervention that eliminates reliance on perfect logistics, documentation, and compliance, thereby ensuring maximum protection for every child.
